# A case report of polymicrobial bacteremia with *Weissella confusa* and comparison of previous treatment for successful recovery with a review of the literature

**DOI:** 10.1099/acmi.0.000119

**Published:** 2020-03-26

**Authors:** Malene Roed Spiegelhauer, Melodi Yusibova, Ida Kirstine Bull Rasmussen, Kristian Asp Fuglsang, Kim Thomsen, Leif Percival Andersen

**Affiliations:** ^1^​ Department of Clinical Microbiology, Copenhagen University Hospital (Rigshospitalet), Henrik Harpestrengs Vej, Copenhagen 2100, Denmark; ^2^​ Department of Gastroenterology and Hepatology, Copenhagen University Hospital (Rigshospitalet), Blegdamsvej 9, 2100 Copenhagen 2100, Denmark

**Keywords:** *Weissella confusa*, bacteremia, case report, review, infection, polymicrobial

## Abstract

*
Weissella confusa* is a Gram-positive coccus and a commensal bacterium of the human gastrointestinal tract with a potential to cause invasive infections. We report the presence of *W. confusa* in the blood of a 25-year-old male patient with Crohn’s disease, short bowel syndrome treated with home parenteral nutrition, and a history of recurrent bloodstream infections, admitted to our hospital with fever and malaise. A polymicrobial culture of *W. confusa* and *Aeromonas hydrophila* was identified from blood, for which treatment with meropenem and metronidazole was initiated. The literature was searched for previous cases of infection with *W. confusa *. In total, 14 reports describing infection of 28 patients were found, most cases presenting with bacteremia. The previous reports have described variable susceptibility to antibiotics; however, all were reported to be vancomycin resistant. Because of its similarities to other vancomycin-resistant cocci, isolates of *W. confusa* might be difficult to identify with traditional methods. Infection may be facilitated by its natural vancomycin resistance, leading to severe infection in hosts with underlying diseases. We describe the treatment of previous cases of infection and suggest treatment methods shown effective in other cases. Vancomycin is often used as treatment of infection with Gram-positive organisms, but this may need to be reevaluated, as several pathogenic bacteria are intrinsically vancomycin resistant. A review on reported treatments of bacteremia by *W. confusa* suggests the use of daptomycin, amoxicillin-clavulanate or piperacillin/tazobactam as recommendable antibiotic regimens.

## Introduction

*Weissella confusa* is a Gram-positive, catalase-negative, nonmotile coccus, which share many characteristics with other lactic acid bacteria [1, 2]. It is facultative anaerobic with a fermentative metabolism and has also been described as part of the group of vancomycin-resistant Gram-positive cocci [3]. It was previously known as *Lactobacillus confusus* and has often been confused with members of the *Leuconostoc *, *Pediococcus* and *Lactobacillus* genera [4]. In 1993, the *Weissella* genus was proposed based on 16S rRNA gene sequencing, and *L. confusus* was reclassified as *W. confusa* [1]. In total, 22 known species of *Weissella* have been described, of which *W. confusa* is most frequently identified in clinical cases [5]. It is found as a commensal in various habitats such as the skin, gut, saliva and milk of animals and humans, but also in soil, plants and vegetables [2]. *W. confusa* has been used for its fermentation functions and shows potential as probiotic supplement [2]. Because *W. confusa* is a member of the normal gut bacteria, the gastrointestinal tract is suspected to be a reservoir for colonization [3].

## Case presentation and identification of the pathogen

A 25-year-old male with Crohn’s disease, short bowel syndrome with 50 cm of small bowel in continuity from the ligament of Treitz, intestinal failure treated with home parenteral support, and a history of frequent blood stream infections, was admitted to the hospital with fever and malaise. The symptoms debuted only 2 h prior to hospitalization. Physical examination revealed slight abdominal pain and vital signs showed blood pressure 115/63 mmHg, pulse rate 96 beats/min, respiratory rate 20 breaths/min and body temperature 38,4 °C.

Routine blood tests at admission showed elevated concentrations of C-reactive protein at 52 mg l^−1^ (reference: <10 mg l^−1^). Empirical treatment with intravenous antibiotics were initiated, i.e. meropenem 2 g three doses daily, vancomycin 1 g two doses daily and linezolid 600 mg two doses daily, as the patient was allergic to penicillin and fluoroquinolone. The central venous catheter (CVC) was removed 2 h after admission due to several previous cases of sepsis, and instead a temporary non-tunnelled CVC was inserted. Despite these initiatives, the patient developed hypotension, and was transferred to the intensive care unit for inotropic support. In extension an echocardiography was performed and showed no signs of endocarditis. The patient developed intermittent abdominal pain, and consequently the surgeons suspected peritonitis, and added metronidazole to the antibiotic treatment. An ultrasonography and CT scan showed hepatosplenomegaly but did not reveal any infection.

Central blood cultures were drawn at day 1. The aerobic culture grew *Aeromonas hydrophila* and Gram-positive cocci identified as *W. confusa* by MALDI-TOF MS. The *W. confusa* isolate was reported sensitive to gentamicin, clindamycin, imipenem, meropenem and daptomycin, and resistant to penicillin, colistin, erythromycin, ampicillin, tetracycline, cefuroxime, rifampicin, vancomycin, oxacillin, linezolid and moxifloxacin. As both bacterial isolates were sensitive to meropenem, the antibiotic treatment continued with meropenem and metronidazole for 12 days total.

Magnetic resonance imaging of the abdomen performed on day 9 revealed a fistula between the excluded bowel and both the skin and complicated fistula-complex in relation to the bladder and rectum. The patient remained admitted due to recurrence of infectious symptoms, which led to yet another change of CVC at day 16, shortly after the first round of antibiotics were seized. At day 24 a tunnelled catheter was inserted with trouble. Neurological symptoms likely caused by the catheter occurred and a new catheter was inserted on day 39. Due to suspicion of intra-abdominal focus the patient was discharged after a total of 40 days, with additional treatment of cefuroxime 1.5 g/day. Tragically, the patient was found deceased in his home a few days following discharge, due to reasons not related to the infection. This was confirmed by an autopsy. The editor and reviewers have assessed an anonymized version of the pathologist’s summary.

## Literature review

Three databases were searched for the literature in English describing clinical infections with *W. confusa;* REX (The Royal Danish Library, www.rex.kb.dk), PubMed (US National Library of Medicine National Institutes of Health, www.ncbi.nlm.nih.gov/pubmed), and Scopus (Elsevier, www.scopus.com). The search terms used were *Weissella confusa, Lactobacillus confusus*, clinical, infection, bacteremia. The search was last repeated 17 January 2019.

The literature search resulted in 14 publications describing infection with *W. confusa* in 28 patients since the first report in 1990 (Table 1). Reports were most frequent from North America (eight reports) and Asia (four reports), the distribution between sex was 14 males (50 %), 10 females (36 %) and 4 unknown (14 %), and an age group between 4–94 years was represented (median 58 years). Bacteremia was the most frequent infection caused by *W. confusa* and was described in 20 cases (71 % of total), of which 7 patients (39 %) presented with a co-infection [4, 6, 7]. *W. confusa* was also isolated in four cases of diarrhea, two cases of endocarditis, one abscess infection, one osteoarthritis, one cecal carcinoma and one gastrostomy infection [3, 8–12]. The majority of patients recovered after antibiotic treatment, but seven cases were fatal (25 % of total) [6, 9].

**Table 1.** Previous reports of *W. confusa
*

Sex, age (years)	Previous/underlying conditions	Infection type	Co-infection	Treatment	Outcome	Reference
M, 25	Crohn’s disease, short bowel syndrome, intestinal failure, home parenteral support, central venous catheter	Bacteremia	* Aeromonas hydrophila *	Meropenem and metronidazole for 19 days. Discharged with cefuroxime	Survived	This report
F, 63	Crohn’s disease, recurrent gastrointestinal strictures, central venous catheter	Bacteremia	None	Piperacillin/tazobactam	Survived	[13]
F, 94	Knee arthroplasty	Osteoarthritis	None	7 days of levofloxacin for a concomitant *E. coli* bacteremia. The treatment did not clear the knee infection, but no further therapy was pursued.	Survived	[8]
F, 60	Hypertension, aortic intramural hematoma	Bacteremia	None	Teicoplanin and piperacillin-tazobactam for 9 days until discharge from hospital	Survived	[23]
M, 54	Liver transplant recipient, hepatic artery thrombosis, diabetes	Bacteremia	None	Oral metronidazole and oral levofloxacin	Survived	[24]
M, 48	Gastroesophageal adenocarcinoma	Bacteremia	None	Intravenous cefoperazone-sulbactam and metronidazole for 8 days	Survived	[12]
F, 58	Non-Hodgkin’s lymphoma	Bacteremia	*Acinetobacter baumannii, Enterobacter cloacae, Candida albicans,* *Bacillus spp*.	Vancomycin, ceftazidime	Fatal	[6]
M, 68	Chronic obstructive pulmonary disease, pneumonia	Bacteremia	None	Ampicillin-sulbactam	Fatal	
F, 62	B-cell lymphoma	Bacteremia	Methicillin-resistant *Staphylococcus aureus* (MRSA)	Vancomycin, ceftazidime, gentamicin	Fatal	
F.92	Chronic renal failure, vascular dementia	Bacteremia	None	Ampicillin-sulbactam	Fatal	
F, 27	Thyroid goiter, ankylosing spondylitis	Bacteremia	* Escherichia coli *	Amoxicillin-clavulanate	Survived	
F, 62	Ischaemic colitis, non-ST elevation myocardial infarction	Bacteremia	None	No treatment	Fatal	
M, 73	Cancerous peritonitis, asphyxia	Bacteremia	* Chryseobacterium indologenes *	Cefepime	Fatal	
M, 52	Oesophageal cancer	Bacteremia	None	Amoxicillin-clavulanate	Survived	
F, 8	Ileus	Bacteremia	None	Vancomycin, ciprofloxacin, ceftazidime and trimethoprim-sulfamethoxazole	Survived	
M, 64	Subarachnoid hemorrhage	Bacteremia	* Enterobacter aerogenes *	Amoxicillin-clavulanate	Survived	
M, 34	Hematopoietic stem cell transplant recipient	Bacteremia	None	Daptomycin	Survived	[7]
M, 58	Severe burns	Bacteremia	* Enterococcus faecalis *	Daptomycin	Survived	
M, 65	Aortic insufficiency	Infective endocarditis	None	Intravenous penicillin G for 4 weeks and intravenous gentamicin. After 2 weeks, treatment with intravenous moxifloxacin was added for 7 days. Postoperative intravenous cefoperazone for 4 weeks and intravenous gentamicin for 2 weeks	Survived	[11]
M, 4	Peritoneal neuroblastoma	Bacteremia	None	Meropenem, aztreonam, cefoxitin, metronidazole, teicoplanin	Survived	[25]
M, 49	Corticosteroid treatment, chronic alcohol abuse	Bacteremia, endocarditis	None	No treatment	Fatal	[9]
M, 46	Abdominal aortic dissection repair, parenteral nutrition, Hickman catheter, coronary artery bypass grafting,	Bacteremia	* Klebsiella pneumoniae *	Gentamicin and piperacillin-tazobactam for 4 weeks	Survived	[4]
M, 49	None	Abscess infection	None	Cephalotin for 10 days	Survived	[17]
Unknown	Multivisceral transplant recipient	Diarrhea, bacteremia	None	Unknown	Survived	[3]
Unknown	Unknown	Diarrhea	None	Unknown	Survived	
Unknown	Unknown	Diarrhea	None	Unknown	Survived	
Unknown	Unknown	Diarrhea	None	Unknown	Survived	
M, 71	Cecal carcinoma	Routine testing of peritoneal fluid	Polymicrobial	No treatment	Survived	[10]
F, 12	Gastrostomy	Abdominal wall	Polymicrobial	Cephalosporin	Survived	

F: Female; M: Male.

*Weissella* spp. strains have been isolated from various human compartments like blood, skin, infected wounds and faeces [2]. The isolation of *W. confusa* from polymicrobial infections has not allowed a clear proof of signiﬁcance for this species. However, the description of *W. confusa* as a single microbial agent in various infections has demonstrated its role as an opportunistic pathogen. Previous reports have stated that immune-compromised status, presence of a central venous catheter, vancomycin treatment, altered gut flora, recent gastrointestinal procedures and dependency on total parenteral nutrition are suspected to be risk factors for obtaining a *Lactobacillus *-species bacteremia [5, 12, 13]. Since *W. confusa* is closely related to the *Lactobacillus* group, it is possible that the risk factors for infection are similar, and many cases of infection with *W. confusa* are associated with medical procedures prior to the period of infection [8]. In some previous cases, infection with *W. confusa* was suspected to originate as translocation from the gut flora, especially in immune-suppressed patients [12, 13]. It was also suggested that the use of a probiotic supplement might have introduced *W. confusa* to the gut environment, although the bacterial composition of the supplement was not tested [14]. One case identified *W. confusa* in the peritoneal fluid in combination with two other bacterial isolates, however there were no signs of infection, and no clinical significance could be related to this isolate [10]. The cases described in this review only involve reports of *W. confusa* in humans, but a case of septicemia in a foal and systemic infection in a monkey have been described, showing its ability to cause infection in a range of hosts [15, 16].

Several case reports have described infection with *L. confusus,* later classified as *W. confusa* [3, 10, 17]. In a previous study, four strains of *W. confusa* were cultured from the faeces of four children, of which one strain was simultaneously isolated from the blood. Clinical information about these patients was limited [3].

The isolated strains described in the literature were sensitive to a wide range of antibiotics, but all presented resistance towards vancomycin (Table 2). In a previous study, 13 strains of *W. confusa* were isolated from the stool of asymptomatic pediatric liver transplant recipients, thus the strains were believed to be part of their stool microbiota. The isolates showed sensitivity towards ampicillin, daptomycin and teicoplanin, and resistance to vancomycin [18]. Another study found vancomycin-resistant *W. confusa* in faeces from patients, which had not previously been treated with vancomycin, suggesting that the species is intrinsic vancomycin-resistant [3].

**Table 2.** Antibiotic susceptibility of *W. confusa* in previous reports

Antibiotic	Susceptible isolates/total no. of isolates	References
**Aminoglycosides** (gentamicin)	2/2	[8, 17]
**Amphenicols** (chloramphenicol)	2/2	[8, 17]
**Antifolates** (trimethoprim-sulfamethoxazole)	1/5	[4, 6, 8, 17, 25]
**Carbapenems** (imipenem)	2/2	[17, 25]
**Cephalosporins** (cephalothin, cefotaxime, cefoxitin, ceftazidime, ceftobiprole, ceftriaxone, cefuroxime)	7/14	[4, 6, 12, 17, 24, 25]
**Cyclic lipopeptides** (daptomycin)	4/4	[5, 7, 8, 12]
**Glycopeptides** (teicoplanin, vancomycin)	9/10	[4, 7–9, 12, 17, 24, 25]
**Lincosamides (c**lindamycin)	5/5	[4, 8, 12, 24, 25]
**Macrolides** (erythromycin)	6/6	[4, 8, 12, 17, 24, 25]
**Nitroimidazoles** (metronidazole)	0/2	[7, 25]
**Oxazolidinones** (linezolid)	2/2	[6, 8]
**Quinolones** (ciprofloxacin, levofloxacin, moxifloxacin)	7/9	[4, 7, 8, 17, 24, 25]
**Penicillins** (amoxicillin, amoxicillin-clavulanate, ampicillin, piperacillin/tazobactam)	10/12*	[4, 8, 12, 17, 24, 25]
**Rifamycins** (rifampicin)	0/2	[7, 17]
**Tetracyclines** (tetracycline, tigecycline)	6/6	[4, 6–8, 12]

*One isolate showed intermediate sensitivity.

## Discussion

We describe a case of infection in a 25-year old male patient presenting with bacteremia caused by polymicrobial infections involving *W. confusa *. This is the 21st report of bacteremia involving *W. confusa *.

Because of its similarities to closely related bacteria, *W. confusa* may be difficult to identify by traditional morphological and metabolic properties [19]. Analysis of the protein composition with MALDI-TOF MS is increasingly used for identification of bacteria from clinical specimens, but this method may also present difficulties in distinguishing closely related species. In a study by Fairfax *et al*., MALDI-TOF MS successfully identified two isolates of *W. confusa *, and the authors concluded that this method is reliable [20]. 16S rRNA gene sequencing has been described as the most reliable standard for identification and was suggested to ensure a valid identification [7]. However, due to the high similarity of the 16S rRNA gene between species of *Weissella *, sequencing of only a part of the gene may result in misidentification [19]. A PCR described by Fusco *et al*. uses a specific genetic marker only found in *W. confusa* for identification of this species [19]. The use of this method might be considered for use in clinical cases with Gram-positive cocci where a sure identification is not possible with the commonly used methods. A study by Kulwichit *et al*. analyzed 26 clinical isolates of catalase-negative gram-positive cocci with different widely used methods. Four isolates were identified as *W. confusa* by 16S rRNA gene sequencing, even though they were identified as *Streptococcus* spp., *Leuconostoc* spp. and *Lactobacillus* spp. by other methods and commercial kits [21]. Recurrent isolation of vancomycin-resistant Gram-positive cocci from blood cultures should be a cause of suspicion for *W. confusa* infection [4].

Infection with *W. confusa* is suspected to be facilitated by its natural vancomycin resistance in hosts with a compromised immune system or underlying diseases [2]. Treatment with vancomycin is often used when Gram-positive organisms are isolated from the blood of immunocompromised patients. This approach may need to be reevaluated, as several potentially pathogenic bacteria have a natural resistance towards vancomycin [7]. Thus, it is crucial that empirical vancomycin treatment is combined with antibiotics active against Gram-positive pathogens inherently resistant to vancomycin. This could be penicillin, clindamycin, daptomycin, erythromycin and fluoroquinolones which have been used previously to successfully treat infections with the vancomycin-resistant *W. confusa* [8]. In our case, treatment with meropenem was successful. Vancomycin was initially added as empirical treatment to follow the guidelines for catheter infections and remained in the treatment regimen to prevent subsequent infection of various *Enterococcus* species, as the patient presented with abdominal complications. The treatment efficiency for bacteremia with *W. confusa* in previous reports was 67 % (12 survived, 7 were fatal). The successful treatments included daptomycin, amoxicillin-clavulanate, piperacillin/tazobactam in combination with teicoplanin or gentamicin, and metronidazole in combination with levofloxacin, cefoperazone-sulbactam, meropenem, aztreonam, cefoxitin or teicoplanin. The fatal cases were treated with cefepime, ampicillin-sulbactam or vancomycin in combination with ceftazidime or gentamicin, and these are not recommended for treatment. Fortunately, several therapeutic agents encompass *in vitro* activity against *W. confusa *, for instance penicillins, clindamycin, fluoroquinolones, aminoglycosides, carbapenems, linezolid, tigecycline and daptomycin [22]. Thus, the drug of choice must be based on careful antimicrobial susceptibility testing, pharmacokinetic properties and accordingly the site of infection. We suggest the use of meropenem, daptomycin, amoxicillin-clavulanate, or piperacillin/tazobactam for treatment of bacteremia with *W. confusa *. In our case, initial treatment with meropenem, and later with clindamycin was successful.

The use of probiotics was in one case suggested as the source of infection, although *W. confusa* was not isolated from the supplement [13]. Some species previously described as probiotic bacteria (such as *Lactobacillus spp*. or *Leuconostoc* spp.) are increasingly recognized as opportunistic pathogens, particularly in patients presenting with diabetes, cancer or prolonged antibiotic treatment [11]. In patients with intestinal malignancies, reduced integrity of the intestinal epithelial barrier may cause leakage of gut bacteria to the bloodstream and result in invasive infection.

In conclusion, we describe the 21st case of bacteremia caused by a polymicrobial infection involving *W. confusa* in a patient with Crohn’s disease and short bowel syndrome first treated with meropenem and the relapse successfully treated with clindamycin. The isolate was cultured from the blood and identified by MALDI-TOF MS. Furthermore, a review on treatment efficiency for bacteremia with *W. confusa was conducted revealing* the use of daptomycin, amoxicillin-clavulanate, or piperacillin/tazobactam as the most recommendable treatment options.
